# Patient characteristics, procedure details including catheter devices, and complications of catheter ablation for ventricular tachycardia: a nationwide observational study

**DOI:** 10.1002/joa3.12356

**Published:** 2020-05-05

**Authors:** Takeshi Kitamura, Mikio Nakajima, Iwanari Kawamura, Hiroyuki Ohbe, Yusuke Sasabuchi, Hiroki Matsui, Kiyohide Fushimi, Seiji Fukamizu, Hideo Yasunaga

**Affiliations:** ^1^ Department of Cardiology Tokyo Metropolitan Hiroo Hospital Tokyo Japan; ^2^ Emergency and Critical Care Center Tokyo Metropolitan Hiroo Hospital Tokyo Japan; ^3^ Department of Clinical Epidemiology and Health Economics School of Public Health The University of Tokyo Tokyo Japan; ^4^ Data Science Center Jichi Medical University Tochigi Japan; ^5^ Department of Health Policy and Informatics Tokyo Medical and Dental University Graduate School of Medicine Tokyo Japan

**Keywords:** catheter ablation, Diagnosis Procedure Combination database, nationwide observational study, ventricular tachycardia

## Abstract

**Background:**

Nationwide data are insufficient with respect to the characteristics of patients undergoing ventricular tachycardia (VT) ablation, complications of VT ablation, and procedure details including catheter devices used during VT ablation. The present study was performed to describe the patient characteristics, procedure details including catheter devices, and in‐hospital complications of catheter ablation for VT using a national inpatient database.

**Methods:**

We used the Diagnosis Procedure Combination database, a national Japanese inpatient database, to identify patients who underwent VT ablation from July 2010 to March 2017. We examined patients’ age, gender, baseline diseases, comorbid conditions, admission status, catheter devices and drugs used, and in‐hospital complications of VT ablation.

**Results:**

We identified 10 641 patients (median age, 61 years) who underwent VT ablation. The most frequently observed background heart disease among patients with structural heart disease was ischemic cardiomyopathy. An irrigated ablation catheter was used in 73% of patients, a force‐sensing ablation catheter was used in 22%, and intracardiac echocardiography was used in 25%. The frequency of using these procedures continuously increased over time. Overall, the prevalence of in‐hospital complications was 3.5% (cardiac tamponade, 0.8%; stroke, 0.6%; critical bleeding, 1.9%; mechanical circulatory support, 0.9%; and in‐hospital death, 0.8%).

**Conclusions:**

The results of this study show the clinical features of VT ablation in a real‐world clinical setting. The use of irrigated catheters, force‐sensing catheters, and intracardiac echocardiography increased over time. The prevalence of in‐hospital complications was 3.5%.

## Graphical abstract

In the present study, we evaluated the patient characteristics, procedure details and complications of catheter ablation for ventricular tachycardia from the Japanese nationwide inpatient database. We identified over 10 000 initial VT ablation procedures during 6 years in Japan. We first demonstrated the details of catheter devices used during the procedure in this large‐scale database.

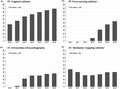

## 1 INTRODUCTION

Ventricular tachycardia (VT) ablation can reduce recurrence of VT and the appropriate therapies by an implantable cardioverter defibrillator.^1^, ^2^, ^3^, ^4^ During the past several decades, the number of VT ablation procedures has been increasing.^5^ New technologies have been introduced and are being frequently used.

However, clinical trials regarding VT ablation are scarce. Most previous studies on VT ablation were based on single‐center experiences from high‐volume centers or multicenter reports of a limited number of patients,^6^, ^7^, ^8^, ^9^, ^10^, ^11^ and their findings may not be generalized to real‐world clinical practice, particularly with respect to the catheter devices used in VT ablation. To our knowledge, no nationwide data are available on the catheter devices used for VT ablation.^5^, ^12^ In addition, most previous large studies focused on VT ablation procedures without a force‐sensing catheter or intracardiac echocardiogram incorporating a three‐dimensional mapping system. A detailed nationwide description of the catheter devices used in VT ablation would be useful to elucidate the current circumstances of VT ablation.

The present study was performed to describe the patient characteristics, procedure details including catheter devices, and in‐hospital complications in patients undergoing VT ablation using a national inpatient database in Japan.

## 2 MATERIALS AND METHODS

The Institutional Review Board of The University of Tokyo approved the present study. The requirement for informed consent was waived because of the anonymous nature of the data.

### 2.1 Data source

We used the Japanese Diagnosis Procedure Combination inpatient database, which includes discharge abstracts and administrative claims data from more than 1200 acute‐care hospitals in Japan. The database includes data on age, gender, diagnoses, comorbidities on admission, complications after admission, procedures including devices used during hospitalization, prescriptions, admission to the intensive care unit, and discharge status (discharge to home, discharge to another facility, and in‐hospital death). Diagnoses were recorded with the International Classification of Diseases, Tenth Revision (ICD‐10) codes along with text data entered in Japanese. Several studies in which this database was analyzed have been reported in the cardiovascular research field.^13^, ^14^

### 2.2 Patient selection

We retrospectively identified patients who underwent an initial ablation procedure for VT (ICD‐10 code I472) and were discharged from April 2011 to March 2017. We excluded patients aged <18 years and those patients who underwent VT ablation more than once during the same hospitalization.

### 2.3 Patient characteristics and procedure details

The patient characteristics evaluated in this study were age, gender, structural heart disease (SHD) (ICD‐10 codes I20–25, I420, I428, I421, I422, I423, I424, I425, I426, I427, I429, I43, I11, I13, and Q2), ischemic cardiomyopathy (I20–25), nonischemic cardiomyopathy^15^ (I420, I428, I421, I422, I423, I424, I425, I426, I427, I429, I43, I11, I13, and Q2), dilated cardiomyopathy^16^ (I420), and arrhythmogenic right ventricular cardiomyopathy^17^ (I428).

The comorbidities evaluated were diabetes mellitus (E100–149),^18^, ^19^ chronic kidney disease,^18^, ^19^ and congestive heart failure. Chronic kidney disease was defined as a recorded diagnosis of ICD‐10 code N18, N19, I120, I131, N032‐037, N052‐57, N250, Z490‐492, Z940, or Z992 or a requirement for renal replacement therapy. Diagnoses of congestive heart failure in this database have been previously validated.^20^

We also assessed the prevalence of urgent admission and intensive care unit admission before ablation. We evaluated the following procedures and drugs before VT ablation: mechanical ventilation, circulatory mechanical support, cardiopulmonary resuscitation, intravenous amiodarone, intravenous nicorandil, intravenous carperitide, intravenous diuretics, intravenous nitrates, beta blockers, diuretics, amiodarone, aldosterone antagonist, and other antiarrhythmic drugs.

We examined the procedure details of VT ablation, including use of general anesthesia, use of an irrigated ablation catheter, use of a force‐sensing ablation catheter, use of intracardiac echocardiography (we only extracted data on intracardiac echocardiography incorporating a three‐dimensional mapping system), and use of a multipolar mapping catheter. Furthermore, the annual trends in the use of each device were investigated. We showed the percentage of each device used per annual number of VT ablation procedures performed.

### 2.4 Complications

The following complications after VT ablation were investigated: cardiac tamponade requiring pericardiocentesis, open chest surgery, open chest removal of hematomas, pericardial sutures, or pericardial incision; stroke identified with an ICD‐10‐based diagnosis during hospitalization (I630–635, I638, or I639); critical bleeding requiring blood transfusion; use of mechanical circulatory support; and in‐hospital death.

We showed the annual prevalence of complications using the above five variables. After tallying the number of patients who underwent VT ablation on an annual basis, the prevalence of complications per year was calculated.

### 2.5 Statistical analysis

Categorical variables are reported as numbers and percentages, and continuous variables are reported as median with interquartile range. We compared patient characteristics and the prevalence of total complications between patients with and without SHD using the Wilcoxon rank‐sum test or Fisher's exact test. To assess annual trends, we used the Cochrane‐Armitage test for proportions. All analyses were performed using Stata/MP 15 (StataCorp).

## 3 RESULTS

### 3.1 Patient characteristics

**FIGURE 1:**
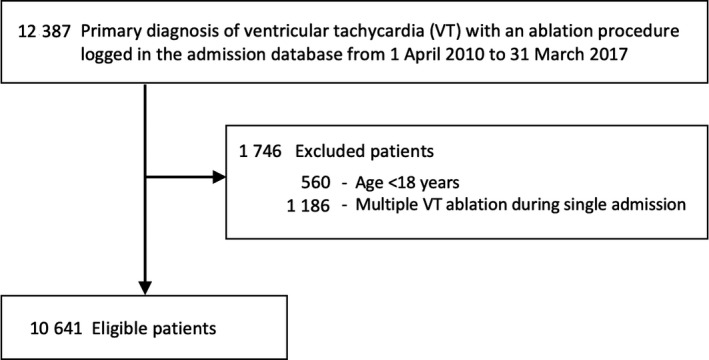
Patient selection. ICD, International Classification of Diseases; VT, ventricular tachycardia

We enrolled 10 641 patients who underwent VT ablation (Figure 1). Table 1 summarizes the patients’ characteristics. The median age of the patients was 61 years (interquartile range, 48–70 years). A total of 4276 (40%) patients had SHD. The most frequently observed comorbidity was ischemic cardiomyopathy (27%).

**TABLE 1:** Baseline characteristics of patients who underwent ventricular tachycardia ablation

Variables	No. of patients (n = 10 641)	(%)
Age, years, median (IQR)	61	(48, 70)
Male	7039	(66.1)
Structural heart disease	4276	(40.2)
Ischemic cardiomyopathy	2895	(27.2)
Nonischemic cardiomyopathy	1381	(13.0)
Dilated cardiomyopathy	685	(6.4)
ARVC	61	(0.6)
Comorbidities		
Diabetes mellitus	1691	(15.9)
Chronic kidney disease	318	(3.0)
Renal replacement therapy	161	(1.5)
Congestive heart failure	3945	(37.1)
Urgent admission	4344	(40.8)
ICU admission before ablation	1213	(11.4)
Procedures before VT ablation		
Mechanical ventilation	331	(3.1)
Circulatory mechanical support	64	(0.6)
Cardiopulmonary resuscitation	75	(0.7)
Intravenous drugs before VT ablation		
Intravenous amiodarone	893	(8.4)
Intravenous nicorandil	157	(1.5)
Intravenous carperitide	284	(2.7)
Intravenous diuretics	499	(4.7)
Intravenous nitrates	1014	(9.5)
Oral medication therapy before VT ablation		
Beta blockers	5240	(49.2)
Diuretics	2758	(25.9)
Amiodarone	2687	(25.3)
Aldosterone antagonist	1869	(17.6)
Other antiarrhythmic drugs	3073	(28.8)

Note

Data are shown as number (%) unless otherwise specified.

Abbreviations: IQR, interquartile range; ARVC, arrhythmogenic right ventricular cardiomyopathy; ICU, intensive care unit; VT, ventricular tachycardia.

### 3.2 Procedure details of catheter ablation for VT

**FIGURE 2:**
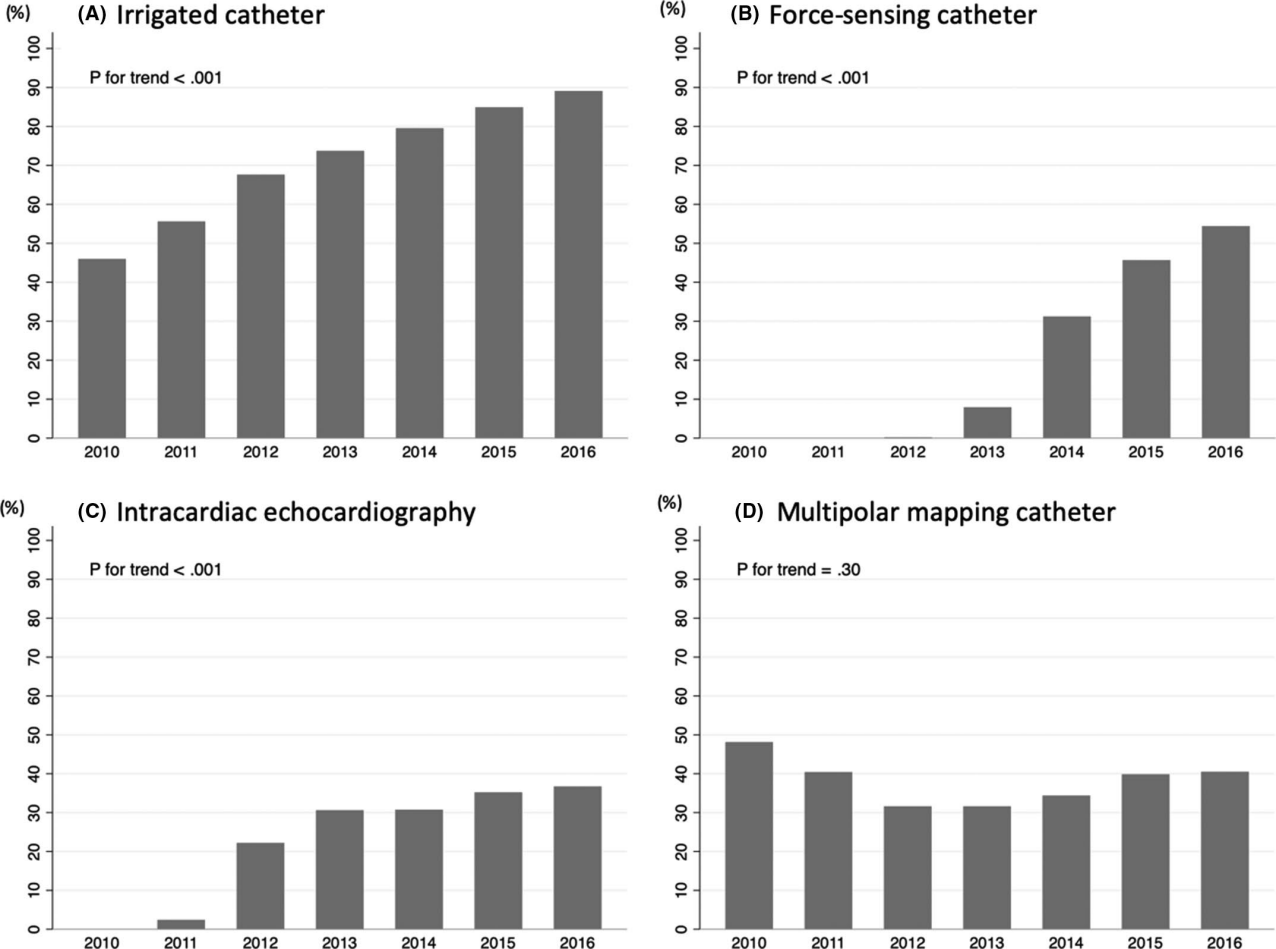
Trends of catheter devices used for ventricular tachycardia ablation

In total, 310 (2.9%) patients underwent general anesthesia, 7774 (73%) were treated with an irrigated ablation catheter, 2339 (22%) were treated with a force‐sensing ablation catheter, 2612 (25%) underwent intracardiac echocardiography, and 3975 (37%) were treated with a multipolar mapping catheter (Table 2). The trend of use of each device is shown in Figure 2. The prevalence of annual use of irrigated ablation catheter, force‐sensing catheter, and intracardiac echocardiography except for multipolar mapping catheter increased over time.

**TABLE 2:** Procedure details of ventricular tachycardia ablation

Variables	Number of patients (n = 10 641)	(%)
General anesthesia	310	(2.9)
Irrigation catheter use	7774	(73.1)
Force‐sensing catheter use	2339	(22)
Intracardiac echo use	2612	(24.5)
Multipolar mapping catheter use	3975	(37.4)

### 3.3 Complications

Table 3 summarizes the ratio of complications. In total, 0.8% patients had cardiac tamponade, 0.6% had stroke, 1.9% had critical bleeding, and 0.9% required mechanical circulatory support after VT ablation. The in‐hospital mortality rate was 0.8%.

**TABLE 3:** Complications in patients who underwent ventricular tachycardia ablation

Variables, n (%)	Total (n = 10 641)		Non‐SHD (n = 6582)		SHD (n = 4059)		*P*‐value
Total major complications	374	(3.5)	152	(2.3)	222	(5.5)	<.001
Cardiac tamponade	83	(0.8)	49	(0.7)	34	(0.8)	.60
Stroke	59	(0.6)	26	(0.4)	33	(0.8)	.005
Critical bleeding	206	(1.9)	76	(1.2)	130	(3.2)	<.001
Mechanical circulatory support	96	(0.9)	27	(0.4)	69	(1.7)	<.001
In‐hospital death	80	(0.8)	21	(0.3)	59	(1.5)	<.001

Abbreviation: SHD, structural heart disease.

Figure S1 demonstrates the annual trend of total complications. From 2010 to 2016, the prevalence of annual complications failed to show any signs of significant fluctuation (*P* for trend .07).

### 3.4 Comparison of patients with and without structural heart disease

Table S1 shows the characteristics of patients with or without SHD. Patient backgrounds significantly differed between the two groups among all variables. In addition, Table 3 demonstrates the prevalence of in‐hospital complications between the two groups. The prevalence of complications was significantly higher in patients with SHD than that in patients without, with the exception of cardiac tamponade.

## 4 DISCUSSION

We identified more than 10 000 initial VT ablation procedures during the 6‐year study period in Japan. In this study, VT ablation in patients with SHD accounted for nearly 40% of all ablation procedures. This is the first study to demonstrate the details of catheter devices used during the procedure in a nationwide large‐scale database. The prevalence of overall complications was 5.0%, and the in‐hospital mortality rate following the procedure was 0.8%.

Regarding the characteristics of patients undergoing VT ablation, two nationwide studies have provided real‐world data of VT ablation.^5^, ^12^ The present study included a cohort of patients with a median age of 61 years, which is similar to that in a previous nationwide study in the United States (US) from 1994 to 2011 ^12^. A more recent US study, however, included patients with a mean age of 70 years from 2000 to 2012.^5^ The proportion of patients with SHD was approximately 40% in the present study, which is similar to that in a previous US study (32.6%); in the more recent US study, however, it was 75.8%. The reason for this difference remains unclear, but we speculate that VT ablations have recently been more likely to be performed for older patients in the US.

Detailed data on catheter devices were not available in previous nationwide or large‐scale studies of VT ablation.^5^, ^12^, ^18^, ^21^ The present study is the first to demonstrate that the use of irrigated catheters increased year by year. This may be because in a previous study, irrigated catheter use was associated with favorable outcomes particularly in patients with SHD.^6^ The use of force‐sensing catheters and intracardiac echocardiography also increased with time, possibly because of the potential advantages of both devices.^22^, ^23^ However, no studies have shown the clinical benefits of both devices in terms of the efficacy and safety of VT ablation.^23^ Our results will encourage further research to assess the efficacy and safety of VT ablations with a force‐sensing catheter or intracardiac echocardiography.

VT ablation can be a challenging procedure and is associated with a certain risk of complications.^5^, ^6^, ^7^, ^8^, ^9^, ^10^, ^11^, ^12^, ^18^, ^21^ In previous studies, the complication rate after VT ablation ranged from 3.3% to 11.2%.^5^, ^6^, ^7^, ^8^, ^9^, ^10^, ^11^, ^12^, ^18^, ^21^ Our findings are comparable to the findings of these previous studies. The postprocedural in‐hospital mortality rate in our study (0.8%) was lower than those in previous studies (1.1%‐2.7%).^8^, ^12^, ^21^ This discrepancy may be caused by differences in study populations and periods.

Although a nationwide study conducted in the US^12^ showed that the prevalence of complications increased over time and in a concurrent manner with an increasing number of VT ablation procedures, our data demonstrated the prevalence of complications did not fluctuate significantly over time. In the US study, they speculated that the increased success rate and operator competence may have contributed to operators tackling more severe cases, which may have inadvertently led to the increased number of complications reflected in the annual trend.^12^ Generally, improved procedural technique and technologies may have the potential to offset some of the risks inherent to VT ablation in severe cases. In the present study, multiple catheter devices were increasingly used, which may have influenced the trend of complications observed.

With regard to the comparative analysis between patients with or without underlying SHD, patient backgrounds differed considerably as expected. The general consensus among experts is the risk of complications is lower when VT ablation is performed in patients without underlying SHD as compared to those with SHD.^1^, ^11^, ^12^ The results yielded in the present study are consistent with this consensus and previous studies.^1^, ^11^, ^12^

Our study had several limitations. First, our database lacked data on unmeasured factors such as cardiac function, the ablation strategy, and therapeutic effects of the procedure. Second, detailed data on the VT substrate or VT origin were not available. Third, the lack of data on left ventricular functions precluded risk adjustment for comparing the outcomes between the groups. Therefore, concerning the lack of data above, we did not perform regression analysis to evaluate factors associated with the outcomes. Finally, this study was conducted only in Japan; thus, the generalizability to other countries is limited.

## 5 CONCLUSIONS

The present study showed the patient characteristics, complications, and procedure details including catheter devices used in a real‐world population undergoing VT ablation in the era of widely available modern technologies. Most notably, the use of irrigated catheters, force‐sensing catheters, and intracardiac echocardiography increased over time. Further investigations of the efficacy and safety of VT ablations with these technologies are warranted.

## DISCLOSURE

All authors declare that they have no conflicts of interest.

## Supporting information

Supplementary Material

Table S1
