# Supplementary figures and images for: Patient characteristics, procedure details including catheter devices, and complications of catheter ablation for ventricular tachycardia: a nationwide observational study
Source: J Arrhythm. 2020 May 5;36(3):464–70. doi: 10.1002/joa3.12356 (PMC7279962; doi:10.1002/joa3.12356)

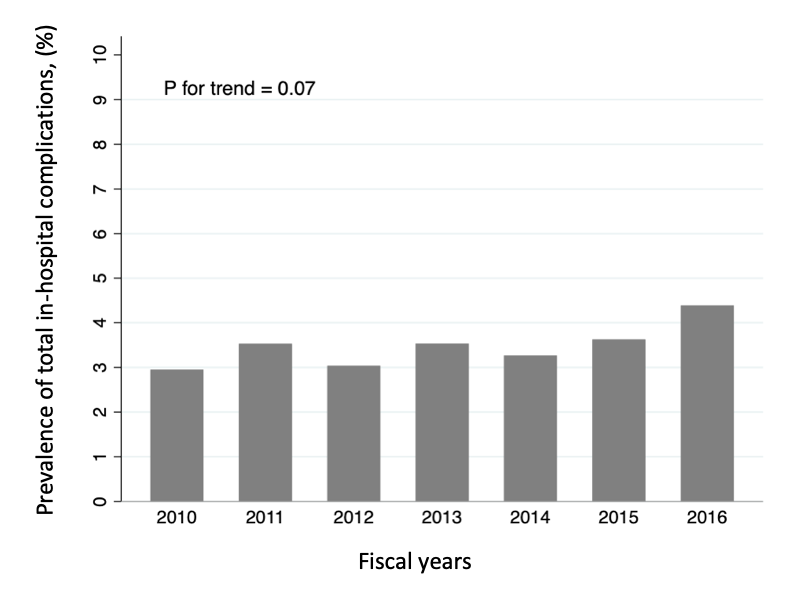

Supplement: Supplementary file 1 — Supplementary Material [file JOA3-36-464-s001.tiff]
