# Supplementary material for: Patient characteristics, procedure details including catheter devices, and complications of catheter ablation for ventricular tachycardia: a nationwide observational study
Source: J Arrhythm. 2020 May 5;36(3):464–70. doi: 10.1002/joa3.12356 (PMC7279962; doi:10.1002/joa3.12356)
Supplement: Supplementary file 2 — Table S1 [file JOA3-36-464-s002.docx]

**Supplementary Table 1.** Baseline characteristics of patients with and without structural heart disease who underwent ventricular tachycardia ablation.

|  | Non-SHD | | SHD | | p-value |
| --- | --- | --- | --- | --- | --- |
| Variables | (n=6,582) | | (n=4,059) | |  |
| Age, years, median (IQR) | 58 | (44-68) | 65 | (54-72) | < 0.001 |
| Male | 3,866 | (58.7) | 3,173 | (78.2) | < 0.001 |
| Structural heart disease |  |  | 4,059 | (100.0) |  |
| Ischemic cardiomyopathy |  |  | 2,895 | (71.3) |  |
| Non-ischemic cardiomyopathy |  |  | 1,381 | (34.0) |  |
| Dilated cardiomyopathy |  |  | 685 | (16.9) |  |
| ARVC |  |  | 61 | (1.5) |  |
| Comorbidities |  |  |  |  |  |
| Diabetes mellitus | 822 | (12.5) | 869 | (21.4) | < 0.001 |
| Chronic kidney disease | 152 | (2.3) | 166 | (4.1) | < 0.001 |
| Renal replacement therapy | 75 | (1.1) | 86 | (2.1) | < 0.001 |
| Congestive heart failure | 1,852 | (28.1) | 2,093 | (51.6) | < 0.001 |
| Urgent admission | 1,287 | (19.6) | 1,298 | (32.0) | < 0.001 |
| ICU admission before ablation | 566 | (8.6) | 647 | (15.9) | < 0.001 |
| Procedures before VT ablation |  |  |  |  |  |
| Mechanical ventilation | 125 | (1.9) | 202 | (5.0) | < 0.001 |
| Circulatory mechanical support | 7 | (0.1) | 57 | (1.4) | < 0.001 |
| Cardiopulmonary resuscitation | 29 | (0.4) | 46 | (1.1) | < 0.001 |
| Intravenous drugs before VT ablation | |  |  |  |  |
| Intravenous amiodarone | 347 | (5.3) | 546 | (13.5) | < 0.001 |
| Intravenous nicorandil | 36 | (0.5) | 121 | (3.0) | < 0.001 |
| Intravenous carperitide | 97 | (1.5) | 187 | (4.6) | < 0.001 |
| Intravenous diuretics | 172 | (2.6) | 327 | (8.1) | < 0.001 |
| Intravenous nitrates | 383 | (5.8) | 631 | (15.5) | < 0.001 |
| Oral medication therapy before VT ablation | |  |  |  |  |
| Beta blockers | 2,470 | (37.5) | 2,770 | (68.2) | < 0.001 |
| Diuretics | 1,083 | (16.5) | 1,675 | (41.3) | < 0.001 |
| Amiodarone | 972 | (14.8) | 1,715 | (42.3) | < 0.001 |
| Aldosterone antagonist | 698 | (10.6) | 1,171 | (28.8) | < 0.001 |
| Other anti-arrhythmic drugs | 1,657 | (25.2) | 1,416 | (34.8) | < 0.001 |

Data are shown as number (%) unless otherwise specified.

IQR, interquartile range; ARVC, arrhythmogenic right ventricular cardiomyopathy; ICU, intensive care unit; VT, ventricular tachycardia
